# Civil war recurrence and postwar violence: Toward an integrated research agenda

**DOI:** 10.1177/13540661211006443

**Published:** 2021-04-16

**Authors:** Corinne Bara, Annekatrin Deglow, Sebastian van Baalen

**Affiliations:** Uppsala Universitet, Sweden

**Keywords:** Civil war, civil war recurrence, postwar violence, citation network analysis, literature review, conceptual framework

## Abstract

Violence after civil war is a challenge to sustainable peace. Many armed conflicts today are recurrences of previous wars and much of the literature on violence after war explains why armed groups return to the battlefield. But even if peace prevails, many other types of violence take place in postwar environments. This postwar violence is likewise subject to a growing multidisciplinary literature. Using citation network analysis, we show that research on war recurrence and postwar violence has developed in relative isolation from each other—although these phenomena are interrelated. This compartmentalization leads us to overlook important similarities and differences in the drivers of different forms of violence after war. We demonstrate this by reviewing the literature in both of these closely related fields. While war recurrence and postwar violence share a set of common risk factors, some factors can have opposite effects on the two outcomes. Because these insights only emerge when systematically comparing the two strands of literature, we propose a novel framework for the study of violence after wars that aims at overcoming the compartmentalization of research within these two fields. The framework serves both as a conceptual lens and an analytical tool to categorize and compare different forms of violence after war. We then outline how the framework aids scholars in pursuing an integrated research agenda, with concrete suggestions for research questions that should be studied to expand our understanding of violence after wars.

## Introduction

Violence in the aftermath of civil war is a challenge to sustainable peace.^1^ The Liberians United for Reconciliation and Democracy’s (LURD) attacks on government positions, for example, reignited the Liberian civil war in 1999. The dissidents, a reconstitution of the group that fought Charles Taylor’s government in the first war, sought to replace the president and take control of the country (Call, 2010). Much of the literature on violence in the aftermath of war aims to explain why armed groups like LURD return to the battlefield (see e.g. Quinn et al., 2007; Toft, 2010; Walter, 2004).

Understanding the causes for the recurrence of civil war is of importance for preventing future loss of life, especially since the vast majority of armed conflicts today take place in countries that have already experienced a civil war (Walter, 2015). Yet, focusing only on war recurrence overlooks many other forms of violence in the aftermath of civil war. The Communist Party of Nepal (CPN-M), for instance, adopted a different strategy than LURD after the November 2006 peace agreement. The CPN-M also continued to use violence against the government and civilians in pursuit of political goals, but instead of doing so overtly, the CPN-M mobilized ex-combatants for violence under the guise of the newly created Young Communist League (YCL) (Joshi, 2014). Unlike in Liberia, the YCL’s violence never provoked a government response of such a magnitude that it amounted to what is generally held to constitute civil war recurrence.

The Liberia and Nepal examples highlight an important fact. Despite sharing many similarities, cases like these are often studied in isolation from one another. Cases like Liberia are generally studied by scholars of *civil war recurrence*, which is commonly defined as a return to collective violence between a government and a rebel group over a political incompatibility that reaches a certain level of intensity. Cases like Nepal are of interest to scholars of *postwar violence*—forms of physical violence that take place in a state where an armed conflict has ended but that falls short of war recurrence. Postwar violence has been the subject of a large multidisciplinary literature (for an overview, see Gartner and Kennedy, 2018) that studies the determinants of phenomena such as political violence, violent crime, sexual violence, or ethnic clashes in postwar societies.

This article proceeds in three steps. First, we use citation network analysis to demonstrate that researchers mostly study civil war recurrence and postwar violence in distinct scholarly fields. While a clear distinction between the two phenomena has been key in facilitating empirical research on these respective outcomes, we argue that compartmentalization has also limited our understanding of violence in the aftermath of civil war more broadly.

Second, we demonstrate the pitfalls of compartmentalization by comparing the literature on both of these closely related outcomes. Two key insights emerge from this review. First, civil war recurrence and postwar violence share a set of common risk factors, that is, factors that trigger both the recurrence of civil war and increase the risk of postwar violence. While this is important knowledge—as it points to areas of priority for those who seek to prevent violence in the aftermath of civil war—it has thus far remained hidden. Second, studying civil war recurrence and postwar violence separately obfuscates that some factors have divergent effects on these outcomes. Our review shows that while military victories, international peacekeeping, and power-sharing provisions reduce the risk of civil war recurrence, the same factors can heighten the risk of different forms of postwar violence. This is important knowledge for policymakers who wish to avoid the potential adverse consequences of otherwise effective interventions. Yet again, this has remained hidden as a consequence of compartmentalization.

Third, we seek to promote an integrated research agenda on violence in the aftermath of civil war around these knowledge gaps by providing a novel conceptual framework. Our framework encompasses all forms of physical violence committed after a civil war has been terminated by a negotiated settlement, military victory, or through low intensity. This violence aggregates to a pattern that marks it as a deviation from expected levels, and that is linked to the preceding war in the sense that either the actors that perpetrate or the conditions that foster the violence were created by the armed conflict. Our framework functions as a *conceptual lens* through which distinct forms of violence in the aftermath of war can be classified beyond the simple war recurrence–postwar violence dichotomy. The framework also serves as an *analytical tool*, which can be used to identify and delimit those instances of violence that are similar enough to warrant comparisons, yet are not usually compared. Using this framework, we outline a research agenda that aims to answer the questions that the current compartmentalization of research on war recurrence and postwar violence has left unanswered.

Our contribution is therefore to provide a novel framework that raises new questions that can only be answered by bringing scholarship on war recurrence and postwar violence together. With this, we hope to stimulate debates and trigger novel research that takes a broader perspective on violence in the aftermath of civil war. After all, violence does not just “fall” into different categories. Instead, structural conditions in postwar contexts and actors’ strategic choices determine the outcomes we see. Looking at these conditions and choices in an integrated manner can help us understand the drivers of different forms of violence in the aftermath of war.

The article is structured as follows. In the next section, we map the respective research fields by first outlining conceptual definitions of civil war recurrence and postwar violence as commonly provided in these fields. We then present the citation network analysis that shows that existing work on war recurrence and postwar violence is conducted by distinct scholarly communities. Thereafter, we compare the two strands of literature and show that insights on shared risk factors and divergent effects only become visible when looking at these two fields in tandem. Motivated by these insights, we propose a framework that overcomes the compartmentalization and helps us to outline an integrated research agenda on violence in the aftermath of war.

## Mapping the fields

What is civil war recurrence and postwar violence? There are both similarities and differences in regards to how scholars define these concepts in the existing literature. Both civil war recurrence and postwar violence occur in the postwar period and therefore rely on a differentiation between war and peace. Galtung (1969: 183) defines peace as the absence of both personal and structural violence and observes that even in the absence of violence, “a tremendous amount of variation is possible” (Galtung, 1969: 168). This resonates with scholars of postwar violence who present typologies of different postwar orders or forms of peace (Höglund and Söderberg Kovac, 2010; Steenkamp, 2005; Suhrke, 2012), and scholars who aim to unpack the “peace continuum” (Davenport et al., 2018). While peace is a multifaceted concept that can refer to something more than the absence of violence, the studies that we review here concern the absence of *physical* violence. Moreover, existing studies commonly focus on postwar contexts as environments where an armed conflict has been officially terminated through either a decisive military victory, a negotiated settlement, and/or where the number of battle-related deaths has fallen below a particular threshold (Kreutz, 2010: 244).^2^ Such postwar environments also encompass countries where armed conflicts are “terminated” in the sense that the intensity of violence is low and the conflict is “frozen.”

Civil war recurrence, by most accounts, thus refers to a return to collective violence between a government and a rebel group over a political incompatibility that reaches a certain level of intensity (see e.g. Kreutz, 2010; Walter, 2004: 376). There are four key theoretical dimensions to this general definition. First, there is a *stated political incompatibility*, that is, a disagreement between at least two actors that concerns government or territory (Pettersson and Eck, 2018). Second, the *intensity of violence* is sufficiently high. While there is no agreement on an exact threshold (see e.g. Call, 2012), there is consensus that violence needs to cross some threshold. Oftentimes, this threshold is given by the operationalization in a particular study, such as the 25 battle-related deaths per year used by the Uppsala Conflict Data Program (UCDP) data set (Gleditsch et al., 2002; Pettersson and Öberg, 2020) or the 1,000 deaths used in the Correlates of War (COW) project (Sarkees and Wayman, 2010).^3^ Third, the nonstate actor is an *organized group*, that is, it displays some form of organizational capacity to collectively and purposefully perpetrate violence. And finally, the *state is a party* to the violence, either as a perpetrator, or as a target. Hence, this definition refers to *civil war* recurrence, that is, recurrence of an armed conflict between a government and a nonstate actor, and not the recurrence of armed conflict between either two nonstate actors, or two states.

There is less consensus on the definition of postwar violence (Gartner and Kennedy, 2018: 7–8). However, most scholars draw on an implicit or explicit definition of postwar violence as physical violence that takes place within a context where an armed conflict has ended and that is either perpetrated by actors, or generated by conditions, linked to the civil war. There are three important dimensions to this implicit definition. First, most scholars stipulate that postwar violence takes place within a context where an armed conflict has ended but that in and off itself *does not constitute civil war recurrence* (see e.g. Bara, 2020: 3). Second, postwar violence is often seen as *excess* violence in the sense that it exceeds an implicit (and often not specified) baseline level of intensity (Gartner and Kennedy, 2018: 9–11). In practice, the existing literature often classifies violence in postwar contexts as postwar violence when it is identified as a deviation from the expected level of violence, with common baselines including prewar levels (Archer and Gartner, 1976), comparable postwar contexts (Van Baalen and Höglund, 2019), or simply low levels of any form of violence (Steenkamp, 2011). Third, postwar violence is considered a *legacy of the war*, meaning that either the actors that perpetrate or the conditions that foster the violence were created by the civil war, that is, would not have been present if the war had not taken place. For instance, while organized crime is not unique to postwar societies, violence by organized criminal groups is often considered postwar violence when perpetrated by ex-combatants (Jarman, 2004; Richani, 2010), or when actors with no active involvement in the war tap into opportunities for enrichment that were created by the economies of war. In addition, Steenkamp discusses how postwar violence arises as the result of a “culture of violence, which creates a socially permissive environment within which violence can continue” (2005: 253) after the war has ended.

In line with these conceptualizations, we limit the scope of our paper to cover physical forms of violence that happen after a civil war has been terminated by a negotiated settlement, military victory or through low intensity that aggregate to a pattern that extends beyond isolated instances of violence, and that is linked to the preceding war. This definition can be applied to any relevant subnational unit. Some countries experience several simultaneous armed conflicts, meaning that when one ends others may continue. Thus, the characterization of postwar environments depends on the unit of analysis.

### Citation network analysis

**Figure 1.**
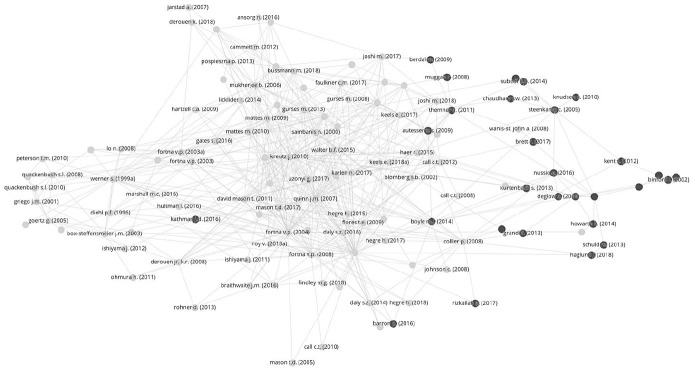
Citation network analysis of research on war recurrence and postwar violence.

A citation network analysis, illustrated in Figure 1, shows that research on violence in the aftermath of civil war is clearly compartmentalized into two distinct fields—civil war recurrence and postwar violence—and that there is little integration of and cross-fertilization between these two fields.

Citation network analysis is one of several bibliometric methods that can reveal a research field’s structure and convey how authors self-organize, intentionally or unintentionally, into certain clusters (Klavans and Boyack, 2017). To evaluate to what extent knowledge is shared across the research fields of civil war recurrence and postwar violence, we conduct a systematic search for publications within each field, combine them into a single database, and analyze the links between them. The sourcing of publications follows a combination of systematic searches for synonym expressions of civil war recurrence and postwar violence, respectively, followed by a manual exclusion of irrelevant search results.^4^ The search is performed in the Scopus database, and sources articles, books, and book chapters published between January 1996 and December 2018. This search yields a sample of 142 publications on civil war recurrence and 106 publications on postwar violence.^5^ The sample is analyzed and visualized using the VOSviewer software (Van Eck and Waltman, 2010).

In the graph in Figure 1, circles represent publications, with the name of the first author and the publication year next to it.^6^ Publications that belong to the civil war recurrence literature are colored in light gray, while publications that belong to the postwar violence literature are dark gray. A link between documents exists when one cites the other. This is visualized as a gray line. In terms of closeness, publications that are closer to each other (clustered) are more closely related in terms of whom they cite and who cites them.^7^

Across both fields, only 163 of the 248 documents are linked to any other document in the sample. In the graph, we only include publications that have a minimum of two links. The rationale for a two-link criterion is straightforward—two is the minimum number of links necessary for a document to be an integrated part of a network. If we think of a tiny network, namely a cluster of three documents that are all related, then each document has to have one link each to the other two, that is, two links in total. Documents with only one link can only be attachments or “tails” at the fringes of integrated networks.^8^ This leaves us with a sample of 130 documents represented in Figure 1.

A first insight that emerges from the citation network analysis is that research on postwar violence does constitute a distinct research field. This was not a given *a priori*. While scholars of civil war recurrence focus on a more or less clearly delineated outcome, scholars of postwar violence study much more diverse outcomes, including postwar crime and homicide, gang violence, political assassinations, civilian targeting, sexual violence, state violence and repression, and communal violence (Gartner and Kennedy, 2018: 3).

Despite this diversity, the closeness of postwar violence publications in the graph and the various links between documents indicate that there is a group of scholars who identify with a larger literature in the way they situate their contribution and review related work. This group is fairly small, especially compared to the much larger group of scholars that work on civil war recurrence. Critics may surmise that our search string fails to capture the diversity of violence studied and that the field is therefore not accurately represented here. While no search string ever captures a field exhaustively, the main reason why the postwar violence cluster is so small is that 25 percent of the postwar violence articles we have sourced have no citation links to any other publication in our sample, and another 16 percent do not have at least two links. This means that although there is a nucleus of scholars who identify as part of a research field on postwar violence, many others doing related work do not, judging from their citation behavior.

The second insight from the network analysis is that civil war recurrence and postwar violence are relatively separate research fields, as indicated by the fact that the lighter and darker circles are largely concentrated on either side of the graph. If authors from both fields would identify as part of a larger literature on violence in the aftermath of civil war and were equally likely to draw on research from either field, the light and dark circles would be more randomly distributed. Also, the different outcomes studied in the two fields are their most distinguishing feature. In other words, there is no cross-cutting theme that unites the fields more than what separates them. Such a cross-cutting theme could be the causes that are studied or different academic traditions and methods. In that case, we would see that there are gaps within the fields that are larger than the gap between them, which is not the case. There is a slightly separate cluster of articles on the left-hand side of the graph, which distinguishes research on inter-state war recurrence from the main cluster of research on civil war recurrence, but the divide between these is less pronounced than the divide between research on war recurrence and postwar violence.

That said, there are citation links between the two fields, suggesting that at least some scholars speak to each other and draw on each other’s work. However, a closer look reveals that this is a one-sided affair. While about two thirds of the postwar violence scholars in our sample have citation links to the civil war recurrence field, the same is true for only a fifth of war recurrence scholars. With very few exceptions (less than ten), civil war recurrence scholars do not cite the work of their postwar violence peers. That is, almost all links between the fields come from postwar violence scholars referencing earlier work on war recurrence. They frequently do this to distinguish their contribution from a literature they believe has a too narrow focus, while recurrence scholars “have largely ignored the variation in human security across postwar states” (Kathman and Wood, 2016: 151).

Both the size of the postwar violence network and the one-sided citation behavior described above may be partially explained by the fact that most contributions on postwar violence are more recent. Thus, there is the possibility that the fields will become more integrated as time passes. In addition, the divide we see between the two fields may also be a methodological one. The majority of work on civil war recurrence is quantitative-comparative, while most research on postwar violence consists of qualitative case studies or conceptual contributions. There are a few publications that more frequently cite and are cited in both fields. Themnér (2011) and Boyle (2014) are two examples. This is because the violence they study could, but does not have to, result in civil war recurrence. As such, their research lies at the intersection of these two fields but does not answer the question of why we sometimes see an all-out civil war recurrence while violence at other times continues in different forms in the postwar period.

## The pitfalls of compartmentalization

The compartmentalization of the two research fields is troublesome from both an academic and policy perspective. To demonstrate the pitfalls of compartmentalization, we review the two strands of literature. Rather than providing an exhaustive review, we exemplify why the compartmentalization of the two fields is problematic by focusing on two aspects that have thus far remained hidden.

### Discounting shared risk factors

A first pitfall is that compartmentalization can lead researchers to discount shared risk factors, that is, causes that heighten the risk of *both* civil war recurrence *and* postwar violence. Discounting shared risk factors obstructs the identification and design of policy interventions that can reduce violence after war generally, regardless of how this violence manifests. We demonstrate this pitfall by discussing three factors that have been consistently found to increase both the risk of civil war recurrence and postwar violence: civil war severity, continued presence of armed groups and ex-combatant networks, and poor governance.

First, several studies show that civil wars that are more severe in the sense of having caused more deaths recur more often (Fortna, 2008; Gurses et al., 2008; Mason and Greig, 2017; Quinn et al., 2007) and increase the risk of various forms of postwar violence (Boyle, 2014; Deglow, 2016). Several mechanisms account for this association. Civil war severity may increase the level of animosity between the belligerents, which can reduce the belligerents’ commitment to honor peace agreements (Gurses et al., 2008: 139; Quinn et al., 2007: 178), thereby making war recurrence more likely. This seems to be a particularly pervasive problem after civil wars fought along ethnic lines (Gurses et al., 2008; Kreutz, 2010). Within the postwar violence literature, such a climate of mistrust is explicitly linked to reengagements in violence at both the state, collective, and individual level (Steenkamp, 2005, 2011, 2014). Another mechanism is that severe civil wars are more likely to undermine the capacity of political, social, and economic institutions in a way that enables both violent challenges to the state and several forms of postwar violence (Collier et al., 2003; Deglow, 2016; Richani, 2010; Suhrke, 2012). This seems to be particularly severe for security institutions, such as the police, that may have been party to the conflict and are suddenly expected to provide public security for all citizens in an unbiased way (Deglow, 2016; Richani, 2010), an observation strengthened by the finding that police reforms lessen the risk of civil war recurrence (Ansorg et al., 2016).

Second, multiple studies show that postwar environments where armed actors retain the capacity to use violence or with a large pool of ex-combatants available for remobilization are more prone to experience repeat civil wars (Haer and Böhmelt, 2016; Rudloff and Findley, 2016; Themnér, 2013). Likewise, postwar violence scholars show that the continued presence of armed groups or ex-combatant networks are associated with more severe outbreaks of postwar violence (Daly, 2012, 2016; Kreutz et al., 2012; Nussio and Howe, 2016; Van Baalen and Höglund, 2019). While the precise type of armed groups or ex-combatant networks vary across these studies, all hinge on the idea that greater opportunities for the perpetration of violence can reignite fighting or trigger postwar violence. In the civil war recurrence literature, this mechanism is often used within a rational choice framework, where a capacity for violence exacerbates credible commitment problems (see e.g. Faulkner, 2019: 89; Rudloff and Findley, 2016: 23). Postwar violence scholars also link violence to credible commitment problems, even though they refer to commitment problems *within* rather than *between* armed organizations (Daly, 2014, 2016). Moreover, several scholars have suggested that the mere presence of ex-combatant networks is insufficient for violence to erupt, thus shedding light on how individuals with the incentives and capacity to remobilize ex-combatants are critical for repeat civil war and postwar violence alike (Daly, 2014; Themnér, 2013).

Third, several studies show that features associated with poor governance, such as weak political institutions, undemocratic institutions, and female under-representation increases the risk of civil war recurrence (Hegre and Nygård, 2015; Shair-Rosenfield and Wood, 2017; Walter, 2015). In a similar vein, several studies demonstrate that weak political, social, and economic institutions generate an environment that is conducive to postwar violence (Boyle, 2014; Collier et al., 2003; Deglow, 2016; Richani, 2010; Steenkamp, 2014). One underlying mechanism is that weak institutions exacerbate credible commitment problems between the former belligerents, whereas strong institutions can help belligerents overcome such problems by making commitments to peace more transparent and credible (Walter, 2015: 1245). A second mechanism holds that weak state institutions, and law enforcement institutions in particular, undermine the state’s deterrent effect, which lowers the opportunity costs of engaging in violence against the state or other citizens (Deglow, 2016: 788–789; Gartner and Kennedy, 2018: 28–30; Hegre and Nygård, 2015: 988–989). Finally, a third mechanism suggests that poor governance risks cementing existing grievances or generate new grievances that inspire violence (Boyle, 2014; Steenkamp, 2011; Suhrke, 2012).

In sum, the integrated review demonstrates that civil war recurrence and postwar violence may be subject to comparable causes and driven by similar mechanisms. However, such an identification of shared risk factors is only possible through an integrated analysis of civil war recurrence and postwar violence.

### Neglecting divergent effects

A second pitfall is that compartmentalization may lead scholars to neglect divergent effects, that is, findings that suggest that a certain factor has a pacifying effect on the risk of civil war recurrence, while simultaneously triggering postwar violence, or vice versa. Overlooking divergent effects can have serious consequences because it may cloud the unintended negative consequences of policy interventions designed to reduce the risk of violence overall. We illustrate this with a discussion of three factors that are known to reduce the risk of civil war recurrence, yet have also been shown to increase the risk of certain forms of postwar violence: military victories, peacekeeping, and power-sharing.

A well-established finding is that military victories reduce the risk of civil war recurrence (Flores and Nooruddin, 2009; Fortna, 2008; Kreutz, 2010; Licklider, 1995; Ohmura, 2011; Toft, 2010; Werner and Yuen, 2005),^9^ even though there is some disagreement on whether it makes a difference who wins (see e.g. Gurses and Rost, 2013; Kreutz, 2010; Quinn et al., 2007; Toft, 2010). The underlying mechanism is that decisive military victories destroy the losing side’s capacity to return to the battlefield, at least temporarily (Licklider, 1995: 684; Toft, 2010: 15). There are several important qualifications to the argument that military victories reduce the risk of war recurrence. For instance, the effect seems to be less pronounced when the victorious side includes multiple allied rebel parties (Mason et al., 2011; Zeigler, 2016) and is conditioned by regime type (Mason and Greig, 2017) and political power-sharing agreements (Mukherjee, 2006). With regard to postwar violence, however, the record of military victories is much less benign. In a rare comparative analysis, Boyle (2014) finds that levels of strategic violence are higher after military victories. Suhrke (2012) likewise points out that a so-called victor’s peace can be very violent, with one of the most extreme examples being the violence that engulfed Rwanda after the genocide in 1994. The mechanism that links military victories to postwar violence is, however, different from the mechanism linking it to civil war recurrence. Here, the logic is that the new government needs to (re-)establish control and rid society of threats to the new order, leading to anti-civilian violence. Weak capacity on part of the losing side makes such persecutions possible. This mechanism has been clearly demonstrated for several postwar societies, including Spain (Herreros, 2011), Italy (Grandi, 2013), and Sri Lanka (Höglund and Orjuela, 2011).^10^

Second, many studies show that international peacekeeping decreases the risk of civil war recurrence (Collier et al., 2008; Fortna, 2003, 2004, 2008; Hegre et al., 2018; Hultman et al., 2016; Kreutz, 2010; Mason et al., 2011; Ohmura, 2011; Quinn et al., 2007). Two important rationalist mechanisms are that peacekeeping operations deter the belligerents from using violence and help overcome information asymmetries that lead to bargaining failure. In contrast, scholarship on the effects of peacekeeping on levels of postwar violence is less consistent. Some studies corroborate the violence-reducing effects of peacekeeping and show that it decreases levels of postwar violence, such as one-sided violence against civilians (Kathman and Wood, 2016). Other studies demonstrate that peacekeeping may not necessarily be effective in reducing all forms of postwar violence. Salvatore (2019) finds that larger numbers of armed troops in peace operations actually increase levels of homicide in war and postwar societies. Bara (2020) finds a similar effect for postwar violence perpetrated by new armed groups or armed groups not formally part of the conflict against the government. The continuity in levels of violence paired with a change in the type of violence across war and postwar periods has been documented in several country case studies, including South Africa (Van Baalen and Höglund, 2019), the Democratic Republic of the Congo (DRC) (Autesserre, 2009), Guatemala (McNeish and Rivera, 2012), and Cambodia (Barma, 2012). Interestingly, these studies often identify (implicitly or explicitly) a similar mechanism as studies on peacekeeping and civil war recurrence. While they agree that peacekeepers have a deterrent effect on the actors’ use of violence, these studies argue that this deterrent is not equally effective for all forms of violence and may actually incentivize a turn to types of violence that fall outside the peacekeepers’ mandate (Bara, 2020: 4–5; Salvatore, 2019: 841).

Third, it is well established that power-sharing decreases the risk of civil war recurrence (Derouen et al., 2009; Hartzell, 2009; Mattes and Savun, 2009; Ottoman and Vüllers, 2015), even if this effect can be conditional on the exact design of power-sharing institutions or contextual factors (Cammett and Malesky, 2012; Gates et al., 2016; Mukherjee, 2006; Ohmura, 2011; Pospieszna and Schneider, 2013). Moreover, other provisions that can be conceived as a form of power-sharing, like the holding of elections (Flores and Nooruddin, 2012; Keels, 2017)^11^ and democratic reform (Keels, 2018; Shair-Rosenfield and Wood, 2017; Walter, 2015) can under some conditions lead to more durable peace. The key underlying mechanism behind this association is that power-sharing provisions work as “fear-reducing provisions” that help the peace agreement’s signatories to overcome credible commitment problems (Mattes and Savun, 2009: 740–741). However, a troubling finding is that power-sharing seems to also increase armed actors’ propensity to use various forms of postwar violence. Daly (2014), who refers to this as “the dark side of power-sharing,” shows that while power-sharing deals can be successful in solving elite commitment problems, they also exacerbate commitment problems between commanders and mid-level commanders. Such “betrayed and resentful” officers were, for instance, key in remobilizing soldiers for violence in Colombia after the end of *La Violencia* (Daly, 2014, 2016). Rizkallah (2017) observes a similar dilemma in Lebanon, where political power-sharing proved a viable mechanism for ending the civil war, but also left the militias-turned-parties’ population networks and organizations intact, thereby reducing their start-up costs for violent mobilization for political gain. Likewise, Wilson (2016) shows that even though autonomy—a form of territorial power-sharing—contributed to ending the armed conflict in Assam in Northeastern India, that autonomy also incentivized the rebels to target other ethnic communities and rivaling factions to monopolize access to the war’s spoils. These studies show that power-sharing may actually exacerbate credible commitment problems *within* armed groups, preserve armed actors’ capacity for violence, and generate incentives to undermine efforts at a more encompassing peace.

To conclude, the integrated review reveals that several well-established factors that reduce the risk of civil war recurrence have divergent effects on postwar violence. Again, identifying these divergent effects is only possible when we consider civil war recurrence and postwar violence in tandem.

## Overcoming compartmentalization: a new framework

We have shown how the compartmentalization of civil war recurrence and postwar violence research can be problematic. Below, we suggest a novel framework to overcome this compartmentalization. The framework serves both as a conceptual lens through which different forms of violence in the aftermath of war can be categorized, and an analytical tool with which relevant comparisons and novel research questions can be identified. We outline such questions as part of an integrated research agenda on violence in the aftermath of war in the second part of this section.

### Categorizing violence in the aftermath of war

**Figure 2.**
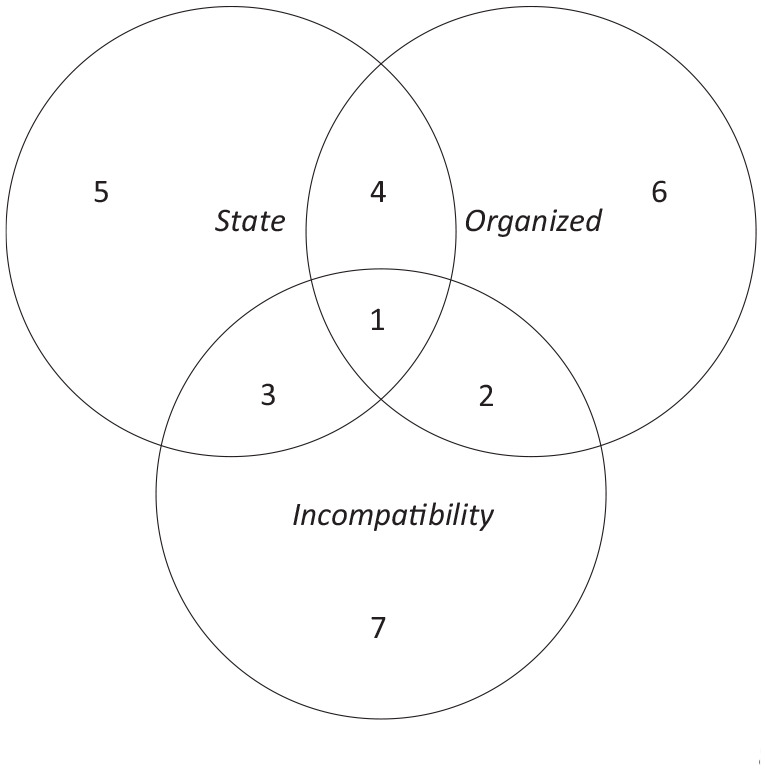
Venn diagram of different types of violence in the aftermath of war.

We begin by outlining how the framework can be used to categorize different forms of violence in the aftermath of war. The framework is visualized in the form of a Venn diagram in Figure 2. The diagram is situated within a rectangle, which sets the basic boundaries for violence in the aftermath of war, which we defined earlier as forms of physical violence that happen after a civil war has been terminated, that aggregate to a pattern that marks it as a deviation from expected levels, and that is linked to the preceding war, meaning that either the actors that perpetrate or the conditions that foster the violence were created by the armed conflict. Within this boundary, the building blocks for the framework are the criteria that are commonly used to define state-based armed conflict, in particular in the UCDP definition (Gleditsch et al., 2002; Pettersson and Öberg, 2020). These are that the state is a party to the conflict, that the nonstate opponent is an organized group, and that the conflict is fought over a clearly stated political incompatibility. We generalize these three criteria to incorporate violence on any level of analysis and use them as categories to classify different forms of violence through their similarities and differences.^12^

The *State* dimension encompasses all violence in which the government is directly involved as a party to the violence, either as perpetrator or target. By government we mean the civil or military branches at the national or subnational level. Violence that takes place between nonstate groups or individuals is excluded from this circle. The *Organized* dimension includes violence that is perpetrated by a formally organized nonstate group. Such a group has a clearly identifiable structure and purpose, and a certain degree of stability and continuity (Kalyvas, 2015: 1519). This includes groups whose main modus operandi is violence and other illegal activities, such as rebel groups or criminal gangs, but also licit organizations like political parties if they use their organization for violence (Sundberg et al., 2012). Violence by individuals or loosely organized or spontaneous groups is excluded here. Finally, the *Political* dimension includes violence with a political purpose. This is the most difficult-to-define criterion when detached from the context of state-based armed conflict, where a political incompatibility exists if an armed challenge to the government aims to either replace the central government or political system, or achieve independence or autonomy for a certain territory (Pettersson, 2020). It is harder to ascertain what “political” means when the state is not involved. While we agree that there are some difficult borderline cases, these are the exception. In most cases where scholars have enough information about the context, it is possible to ascertain whether violence is political in the sense that it is perpetrated by groups who have a publicly articulated disagreement over who governs or how, who has the right to inhabit a territory, or how decision-making over the distribution of public resources ought to function. As should be clear from this definition, ordinary crime—violence perpetrated by and among individuals that are devoid of collective goals—is not political violence. Neither is violence by organized criminal groups, including gangs, mafias, and drug cartels.

Figure 1 uses these three criteria to distinguish different categories of violence in the aftermath of war. Depending on which criteria are fulfilled, we identify eight different categories.

*Insurgency violence* (1) refers to violence that occupies the intersection of all three criteria, thus constituting political violence between a state and a formally organized nonstate armed group. Prime examples of insurgency violence are clashes between government forces and rebel groups, as has been the case in the aftermath of the wars in Liberia (Call, 2010) and Nepal (Joshi, 2014). Since our framework specifies that violence must be aggregating to a pattern that marks a deviation from expected postwar levels, but does not stipulate a strict numerical threshold for the number of violent acts, insurgency violence can encompass both civil war recurrence as commonly defined in quantitative studies, as well as non-lethal or low-intensity insurgency violence.

*Violence between armed groups* (2) is perpetrated by organized nonstate groups for political purposes but lacks direct state involvement. This form of violence took place, for instance, between militia groups in the DRC after 2002 (Autesserre, 2009) and between political parties in postwar Lebanon (Knudsen and Nasser, 2012). Postwar violence between armed groups often resembles a “continuation of war by other means” (Bara, 2020: 980). Many of the violent actors of the war are still active in this violence, either because their outfits where never disbanded, or because they have entered the political arena. This violence is different from a continuation or recurrence of the war, because the state is neither directly targeted, nor officially involved. This does not preclude the possibility that the state is involved as a covert supporting actor, as was the South African state in the violence that hit KwaZulu-Natal after 1990 (Ellis, 1998).

*Violent political unrest* (3) captures political violence between the state and a nonstate actor that is not formally organized as a rebel group or political organization. Common examples of such violence are demonstrations that turn violent, as did the protests that precipitated the 2006 Dili riots in Timor-Leste (Babo-Soares, 2012). Postwar political unrest is often a result of peace deals that leave popular grievances unaddressed or create new ones, or an expression of discontent with the performance of new postwar governments. It can also be instigated by former members of a rebel group to serve their postwar goals (Babo-Soares, 2012; Schuld, 2013). Although the extent to which nonstate actors challenging the government in the streets are organized can be a matter of degree (Jeffery and Hall, 2020; Murunga, 2011), the important distinction is that the nonstate challenger is a more ad hoc and temporary conglomeration of individuals than formally organized nonstate groups.

*Organized criminal violence against the state* (4) is the last form of violence that satisfies two of the three criteria for war recurrence. This is violence between the state and a formally organized nonstate group that makes no political claims on the state, such as a criminal organization. Such violence involves the state, meaning that organized criminal groups battle “not only one another, but the state itself” (Lessing, 2013: 1). Still, organized criminal violence lacks political incompatibility in the sense that financial profit rather than political concessions is the end goal (Kalyvas, 2015: 1520–1521). For postwar contexts specifically, gang violence in El Salvador fits this category (Kurtenbach, 2013). While organized criminal violence often takes place outside civil war contexts (the so-called Mexican Drug War being the most prominent example), postwar societies are particularly prone to this violence. War can create opportunities for illicit economies, and networks forged between belligerents and criminals to finance the war often persist into the postwar period (Gartner and Kennedy, 2018: 31–32). Moreover, weak postwar economies, the demobilization of large numbers of soldiers and/or rebels, or the presence of plentiful firearms facilitate criminal activity (Rivera, 2016).

The next three forms of violence in the aftermath of war only share one characteristic with the type of violence conventionally classified as civil war recurrence, in contrast to the four first categories, which share at least two.

*State violence against civilians* (5) refers to violence committed by a state against civilians where there is no formally organized opponent with a stated incompatibility with the state. There are numerous examples of postwar state violence against civilians, such as the violent repression of ex-combatants in postwar Spain (Herreros, 2011; Richards, 2012) and the reprisal attacks against Hutu civilians following the Rwandan civil war and genocide (Eide, 2012; Kathman and Wood, 2016). While the purpose of this violence from the perpetrator side is often political, for instance by serving to consolidate state power after a civil war (Suhrke, 2012: 8–9), political *incompatibility* would imply two sides who get to articulate their incompatible position, which is not the case when unarmed and unorganized civilians are targeted.

The sixth type is *armed group violence against civilians* (6), which is violence committed by a nonstate actor against civilians (or another nonstate actor) when there is no political incompatibility. Like state violence against civilians, this form of violence lacks a political incompatibility even if the purpose of the violence is political. As Kathman and Wood (2016: 149) note, “factions often persist in abusing civilians to reinforce conflict gains, shape the postconflict environment, exact revenge for wartime grievances, or spoil peace processes.” Two prominent examples are the reprisal attacks carried out against civilians by members of the Kosovo Liberation Army (UCK) after the Kosovo war (Boyle, 2010) and the LTTE’s killing of civilians in Sri Lanka during the 2002–2005 ceasefire (Höglund, 2005). Moreover, this category includes violence between organized criminal groups and by organized criminal groups against civilians, like the violence prevalent in postwar El Salvador (Richani, 2010).

The seventh type of violence is *communal violence* (7), violence that is the product of clashes between nonstate actors that are not formally organized but have a political incompatibility with one another. Most of this violence involves violent clashes between groups that are informally organized along identity lines like religion, ethnicity, or language, and who resort to violence on an ad hoc basis when an incompatibility over power, territory, or resources arises (Von Uexkull and Pettersson, 2018). Communal conflicts and civil wars are often intertwined in that civil wars may arise out of communal conflict, and when they end, violence shifts back to the community level (Brosché and Elfversson 2012; Brosché and Rothbart, 2013). When this happens, the state ceases to be an official party to the conflict. As research shows, however, the state is often indirectly implicated in communal violence by failing to intervene or even instigating it (Brass, 2003; Varshney and Gubler, 2013).

The remaining form of violence in the aftermath of war—represented in the rectangle but outside the Venn diagram—is *interpersonal violence (8).* This violence can be defined as “violence motivated by personal goals or emotions and carried out by individuals, alone or in small groups of friends or acquaintances” (Gartner and Kennedy, 2018: 7). Acts of so-called ordinary crime like murder, assault, rape, and domestic violence fall into this category. As the term “ordinary” signals, this is violence that happens in all societies, even those that have not experienced a civil war in centuries.^13^ As such, this violence primarily catches the attention of postwar scholars when it reaches levels that would have been unlikely if it had not been for the preceding war, as it did in postwar Northern Ireland (Deglow, 2016), Guatemala and El Salvador (Kurtenbach, 2013), northern Uganda (Saile et al., 2014), and the former Yugoslavia (Nikolić-Ristanović, 1998). Because this violence does not share any of the three dimensions with war recurrence in our framework, we have not focused on this violence in either the citation network analysis, or the literature review.^14^ Our framework therefore also distinguishes between collective and interpersonal violence after wars.

### Using the framework for an integrated research agenda

We argue that the framework helps to move scholarship of war recurrence and postwar violence closer together and thereby overcome the pitfalls of compartmentalization. Below, we describe two broad research strategies for an integrated research agenda and clarify how the framework aids researchers who wish to pursue these strategies.

A first strategy is to shift from explaining the causes of outcomes to explaining the outcomes of causes. That is, instead of asking what explains occurrence or one particular form of postwar violence, scholars should explore what forms of violence are associated with a particular risk factor or policy intervention. This strategy can help identify shared risk factors—factors that increase or decrease the potential for violence in the aftermath of war *generally*. Security sector reform (SSR), for instance, could be such a factor. SSR strives to increase the legitimacy of security forces, thereby reducing potential grievances. SSR also strengthens the state’s law enforcement capacity. Berg (2020), for example, shows that robust civilian oversight and a diverse officer corps reduces the risk of civil war recurrence. Similar mechanisms, such as a more professional policing conduct, may also reduce other forms of violence, as it increases citizens’ willingness to rely on and cooperate with police forces.^15^ Yet, to the best of our knowledge, there are no studies that focus on the impact of the same factor on multiple forms of violence in the aftermath of civil war.

In such a research endeavor, our framework can serve as an analytical tool to map the multiple forms of violence that ought to be looked at. The framework can provide a basis for identifying relevant existing research on how a particular risk factor influences various types of violence, and as a starting point for data collection. Moreover, the three dimensions and the way in which they outline similarities and differences between different types of violence can help in *theorizing* about the expected effect of certain factors on different forms of violence in the aftermath of war. For policy, the findings from such an integrated research strategy would be crucial. Knowledge about factors that increase or decrease the risk of violence overall points to areas of priority for those that seek to prevent violence in the aftermath of civil war, whatever form it takes. One question this strategy may not be best suited to answer, however, is why similar risk factors sometimes lead to very different forms of violence across contexts. The second strategy focuses on answering that question.

The second strategy is to compare different types of violence *to each oth*er to explain divergent trajectories of violence across postwar contexts. In particular, we need research that explains why we see an outright war recurrence in some cases, while violence continues in different forms in other cases—forms that are remarkably similar to war recurrence either via the actors involved or the strategic purposes served by the violence. Such a systematic violence comparison strategy is promising for identifying conditions that have opposite effects on different types of violence. We have identified some of these factors in our literature review, but we need a systematic research agenda that studies such inverse relationships. We know, for instance, that armed peacekeeping troops are effective in deterring war recurrence (see e.g. Hultman et al., 2016). However, this effectiveness in altering the strategic environment in which actors operate may come with new and unforeseen challenges, such as an increase in criminal violence (Salvatore, 2019), or a shift toward different forms of violence or actors (Bara, 2020). Such shifts in the form and perpetrators of violence will remain invisible if we continue to primarily compare individual forms of violence to their absence, rather than to related forms of violence. In addition, we miss an opportunity to inform policymakers about potential adverse consequences of otherwise effective interventions.

In pursuing such a research strategy, our framework serves two purposes: First, it helps scholars to systematically identify the most relevant comparison categories. The more dimensions two types of violence have in common, the more important it is to compare them. Second, by clearly indicating on which dimension(s) two types of violence differ, our framework points at the most obvious questions to be answered: Why do we see this difference, and do certain policy interventions contribute to a transformation of violence rather than an overall reduction? Comparing postwar contexts with *insurgency violence (1)* to contexts that see *violence between armed groups (2)*, for instance, stresses the need to examine why the state is not a party to the violence in one case but is in the other. Of course, the state is still often an important player in such violence, either by supporting certain armed groups or even hiring pro-government militia to perpetrate violence on its behalf (Carey and Mitchell, 2017). However, even then this raises the question why the state sometimes participates in violence overtly and sometimes executes violence through proxies. Similar questions could be raised about different outcomes with regard to other forms of violence. For example, comparing cases with *violent political unrest (3)* to cases with *insurgency violence (1)* raises questions about why aggrieved citizens in some postwar contexts are able to perpetrate violence against the state as formal and permanent organizations, while aggrieved citizens in other postwar contexts are not (cf. Walter, 2004, 2015). Thus, a critical aspect of an integrated research agenda on violence in the aftermath of civil war is to focus on comparing, but not conflating, different types of violence in postwar societies. It is not our intention to abandon civil war recurrence or postwar violence as conceptual categories. On the contrary, we envision a research agenda where these categories are seen as part of the same overarching phenomenon—violence in the aftermath of civil war—and serve as a starting point for mapping the various forms of violence present in postwar contexts and identifying relevant comparisons.

## Conclusion

This paper has presented a novel framework to help bridge the gap between research on civil war recurrence and postwar violence, and has outlined how this framework helps to identify precisely what has been obscured by the compartmentalization of the two strands of scholarship: factors that increase both the risk of civil war recurrence and postwar violence as well as factors that decrease the risk of one, but increase the risk of the other. The framework therefore contributes by providing a conceptual lens and analytical tool for scholars to identify a range of research questions that are yet to receive full systematic attention. This, in turn, will increase the policy relevance of scholarly work on postwar environments. While each respective research field has produced important insights, an integrated research agenda as suggested here will expand our knowledge on variations across different forms of postwar violence, thereby helping to identify what policy interventions are best suited to reduce all forms of violence after civil war.

Finally, as an extension, our framework can also be adapted to an integrated study of violence *during* war. Civil wars are defined by insurgency violence reaching a critical severity threshold. Yet, multiple other forms of violence in which a myriad of actors are involved take place in civil war contexts. Rebel groups fight each other, security forces target civilians, militias do the killing for someone else, ethnic groups clash, and gangs fight over illicit business. While this complexity is widely recognized, conflict research mostly studies different forms of political violence in isolation rather than their interrelationships (Findley and Young, 2012; Staniland, 2017). Our framework could therefore be of interest for civil war scholars more generally.

## Supplemental Material

sj-pdf-1-ejt-10.1177_13540661211006443 – Supplemental material for Civil war recurrence and postwar violence: Toward an integrated research agenda Click here for additional data file. Supplemental material, sj-pdf-1-ejt-10.1177_13540661211006443 for Civil war recurrence and postwar violence: Toward an integrated research agenda by Corinne Bara, Annekatrin Deglow and Sebastian van Baalen in European Journal of International Relations

## Notes

1. We use the term violence in the aftermath of civil war as an umbrella term for both civil war recurrence and different forms of postwar violence. These concepts are discussed further in the next section.

2. We acknowledge that the concept “postwar” is strongly influenced by a definition of war that has battle violence between rebels and governments at its core. Yet, when studying different forms of violence that take place in postwar contexts, the boundary between war and peace is blurred. This is an important discussion that is increasingly finding resonance (Campbell et al., 2017; Davenport et al., 2018; Diehl, 2016). At the same time, we do not believe that war and peace, or war and postwar, need to be abandoned as useful conceptual categories. For instance, the end of battles between the main combatants can be a turning point in a history of contention. Likewise, for better or worse, the designation of a context as “postwar” does shape the behavior of policymakers (see e.g. Autesserre, 2009), and is therefore a relevant conceptual category.

3. For a critical discussion of the battle-deaths criterion, see Sambanis (2004).

4. The results are similar if we skip the manual exclusion step. This indicates that the authors of those contributions we do not consider part of either the civil war recurrence or postwar violence field do not consider their contributions part of these fields either, judging from the citation behavior.

5. Scopus has only systematically indexed publications in the social sciences from 1996 onward. We exclude work not in English. More detailed information on the citation network analysis, including search strings, a motivation for why Scopus was preferred over alternatives, and further information on the manual inclusion and exclusion of articles can be found in the Appendix.

6. For technical reasons, we had to remove non-English characters from author names.

7. This is because the VOS algorithm aims to minimize the (weighted) sum of the (squared) overall straight-line distances between linked pairs (Van Eck and Waltman, 2010).

8. A graph showing a citation network based on a one-link criterion can be found in the Appendix. The conclusions we draw on the integration of the two fields do not change.

9. See Hartzell (2009) for contrasting findings.

10. For contrasting findings, see Kurtenbach (2013) and Keels (2018).

11. For a study that suggests that voter turnout increases the risk of civil war recurrence, see Letsa (2017).

12. Note that this framework is meant as a heuristic tool and does not offer strict operational criteria. However, it can serve as a basis for developing such criteria if the aim is data collection and comparison.

13. Though there is nothing “ordinary” about such violence, for legal scholars the ordinary here refers to individual acts that violate domestic criminal law (Aukerman, 2002).

14. Yet, there are good reasons for scholars of collective and/or political violence not to neglect this category. This is because citizens, the state, as well as armed nonstate groups have incentives to reframe political violence as “ordinary violence” after war. This has, for instance, been shown for El Salvador by Moodie (2012) and for Kosovo by Boyle (2014). See Gartner and Kennedy (2018) for a literature review that primarily (but not exclusively) focuses on interpersonal postwar violence.

15. For instance, Blair et al. (2019) find that confidence patrolling increased citizens’ reliance on the formal police in postwar Liberia, and reduced certain forms of crime and domestic violence.
