# Supplementary material for: Civil war recurrence and postwar violence: Toward an integrated research agenda
Source: Eur J Int Relat. 2021 Apr 16;27(3):913–35. doi: 10.1177/13540661211006443 (PMC8427816; doi:10.1177/13540661211006443)
Supplement: sj-pdf-1-ejt-10.1177_13540661211006443 – Supplemental material for Civil war recurrence and postwar violence: Toward an integrated research agenda [file sj-pdf-1-ejt-10.1177_13540661211006443.pdf]

Online Appendix: Civil War Recurrence and Postwar Violence: Towards an Integrated Research Agenda

Corinne Bara, Annekatrin Deglow, and Sebastian van Baalen[1]

Scopus vs. Alternatives

The reason for choosing Elsevier's Scopus database (https://www.scopus.com/) for sampling publications over its main competitor, the Web of Science (WoS) by Clarivate Analytics (https://apps.webofknowledge.com/), was driven by two reasons. First, journal coverage in the social sciences and humanities is broader in the Scopus database than the WoS database (Norris and Oppenheim, 2007). Indeed, many articles that we consider key in either of the two fields, but especially in the field of postwar violence, appeared only in the Scopus search and not in the WoS search. Second, and more importantly, Scopus also indexes books and book chapters, while WoS only indexes journal articles. Kousha and Thelwall (2009) show that we likely miss about 40 percent of all citations in the social sciences if books are not considered, which dovetails with our own experience from trying out both databases. Especially some of the more recently published key works in the postwar violence fields are monographs (Boyle, 2014; Steenkamp, 2014; Suhrke and Berdal, 2012). The only drawback with using the Scopus database is that it only systematically indexes articles in the social science since 1996, but this was considered a minor disadvantage.

Sourcing of publications

Based on our knowledge of the key works in both fields, we create a search term of each literature to retrieve the first set of documents. In the war recurrence field, the search string is designed to retrieve documents in which synonyms of the term 'conflict' appear within three words of synonyms of the term 'recurrence' in either the document title, abstract, or keywords. In addition, we look for the term 'conflict trap' and documents in which 'peace' appears within three words of 'duration'. For the postwar violence field, the search retrieves documents in which synonyms of the term 'postwar' appear within three words of synonyms of the term 'violence.' We then limit the search to publications in the social sciences, humanities, and economics, and to publications written in English. We manually check this procedure to ensure that the key works are captured by the search string. Moreover, in creating the search

[1]Corresponding author: corinne.bara@pcr.uu.se

strings we design them to be sensitive (capture more articles) rather than precise (capture more relevant documents). Because the systematic search is followed by a manual exclusion of documents not deemed relevant, this search strategy yields a more representative overview of the literature in the two respective fields. The search process (before manual exclusion) yields a sample of 545 documents for conflict recurrence and 309 documents for postwar violence.

Search String for Postwar Violence

TITLE-ABS-KEY ((violen* OR crime* OR homicide* OR killing* OR targeting* OR victimization* OR victimisation*) W/3 (postwar OR ”post-war” OR postconflict OR ”post-conflict” OR ”post-agreement” OR ”postagreement” OR postaccord OR ”post-accord” OR postsettlement OR ”post-settlement” OR ”after war*” OR ”after civil war*” OR ”after conflict*” OR ”after civil conflict*” OR ”after armed conflict*” OR ”in the aftermath of war*” OR ”in the aftermath of civil war*” OR ”in the aftermath of conflict*” OR ”in the aftermath of civil conflict*” OR ”in the aftermath of armed conflict*” OR ”after peace agreement*” OR ”after peace accord*” OR ”post-demobilization” OR ”postdemobilization” OR ”post-civil war” OR ”post-armed conflict” OR ”post-civil conflict”)) AND (PUBYEAR >1995 AND PUBYEAR <2019) AND (LIMIT-TO (SUBJAREA , ”SOCI”) OR LIMIT-TO (SUBJAREA , ”ARTS”) OR LIMIT-TO (SUBJAREA , ”ECON”)) AND (LIMIT-TO (DOCTYPE , ”ar”) OR LIMIT-TO (DOCTYPE , ”ch”) OR LIMIT-TO (DOCTYPE , ”bk”) OR LIMIT-TO (DOCTYPE , ”re”) OR LIMIT-TO (DOCTYPE , ”ed”) OR LIMIT-TO (DOCTYPE , ”ip”)) AND (LIMIT-TO (LANGUAGE , ”English”))

Search String for War Recurrence

TITLE-ABS-KEY (((conflict* OR ”war” OR ”wars” OR rebellion* OR insurgenc*) W/3 (recur* OR reoccur* OR renewed OR repeat OR relapse* OR resume*)) OR ”conflict trap” OR (peace W/3 duration)) AND (PUBYEAR >1995 AND PUBYEAR <2019) AND (LIMIT-TO (SUBJAREA , ”SOCI”) OR LIMIT-TO (SUBJAREA , ”ARTS”) OR LIMIT-TO (SUBJAREA , ”ECON”)) AND (LIMIT-TO (DOCTYPE , ”ar”) OR LIMIT-TO (DOCTYPE , ”ch”) OR LIMIT-TO (DOCTYPE , ”re”) OR LIMIT-TO (DOCTYPE , ”bk”) OR LIMIT-TO (DOCTYPE , ”ip”) OR LIMIT-TO (DOCTYPE , ”ed”)) AND (LIMIT-TO (LANGUAGE , ”English”))

Manual Inclusion and Exclusion of Publications

In the next step we manually exclude publications that do not fulfil either of the following three criteria. First, postwar violence or war recurrence has to be the main focus of the

study, that is, the key empirically studied outcome or key focus of a theoretical or conceptual discussion. We are a bit lenient here if a different or more specific outcome (such as community attitudes towards ex-combatants) is studied, but this different outcome is framed as a key factor towards avoiding war recurrence or postwar violence. Second, the "postwar" has to matter. This pertains mainly to documents in the field of postwar violence. A study cannot just happen to be conducted in the postwar period of a war (perhaps because it is easier), but it has to be about the fact that the postwar period matters and what that "does to violence." Third, the violence under examination has to occur after the end of World War II. This criterion mainly serves to ensure the comparability of the two fields. The war recurrence literature has primarily studied the resumption of war post-1946 when the current state system was largely in place.[2] We thus follow the same logic for the literature on postwar violence, which holds somewhat more contributions on pre-World War II cases of postwar violence.

There are, after the manual exclusion, 8 articles that appear in both search strings. Based on the outcome they study, we assign them to one of the two fields (see Table 1). There are also 3 articles that "landed" in the wrong field because of the wording used in their texts, but where from the dependent variable it was clear that they belonged to the other field. We reassigned them accordingly (see Table 1).

Table 1: Overview of duplicate and reassigned articles.

Article	Duplicate or Reassignment	Action
Childers 2014	Duplicate	Assigned to recurrence
Flores and Nooruddin 2009	Duplicate	Assigned to recurrence
Kurtenbach 2013	Duplicate	Assigned to postwar violence
Keels 2017	Duplicate	Assigned to recurrence
Daly 2016	Duplicate	Assigned to recurrence
Brett 2017	Duplicate	Assigned to postwar violence
Mendeloff 2004	Duplicate	Assigned to recurrence
Boyle 2014	Duplicate	Assigned to postwar violence
DeRouen and Chowdhury 2018	Reassignment	From postwar to recurrence
Collier et al. 2019	Reassignment	From postwar to recurrence
Bowsher et al 2018	Reassignment	From recurrence to postwar

There are also a few instances in which the same piece is published twice in different outlets (for instance, once as a book chapter and once as a journal article). In these cases

[2] An example of an exception is Stephen L. Quackenbush (2010), who studies war recurrence between 1816 and 2011.

we unify them into one, meaning: We choose the more frequently cited one, and delete the other, but we make sure that citations to the deleted version are replaced with citations to the non-deleted version so that no citation information is lost.

For ten articles (Berend 2006; Cornell 2014; Joshi 2014; Kreutz et al 2013; Little 2009; Silber 2004; van Baalen and Höglund 2017; Demeritt et al 2014; Jarstad and Sundberg 2007; Sisk 2008) we cannot retrieve the reference list even after checking on other platforms (WoS and the respective journal directly). This happens if articles are still in print (online first); because the document is a book chapter without its own references (references only at the end of the full edited book); or because of a different referencing format (references listed only in footnotes or endnotes and not indexed by Scopus/WoS). In these cases, the article can only be a cited article, not a citing article, which is unfortunate but unavoidable. Edited books also often do not have reference lists in cases in which each chapter has their own reference list, but in these instances we solve the problem by taking the references from the edited books' introductory chapter, which should be fairly representative for the entire book.

Citation Analysis: Specifics

We choose direct citation analysis over the alternative of co-citation analysis. Co-citation analysis is an indirect form of network analysis in which two papers are related if they are cited together by a later third paper. Because many contributions to the postwar violence field were published relatively recently, this emerging literature might no be well reflected in a co-citation analysis. A third alternative is bibliographic coupling, which does not have that age problem. Bibliographic coupling is the "opposite" of co-citation analysis, meaning that two documents are linked if they cite the same third article. Many of these common citations, however, do not reflect a substantive linkage, but rather indicate that the authors use the same data or method and make a reference to a dataset, for instance.

In creating the citation network we do not set a minimum citation threshold as is often done. That is, we do not select a sub-sample of all retrieved publications based on how often a document is cited overall. This is because we do not want to disadvantage more recent publications that are not yet cited as often. In our case, this would bias the results given that the postwar violence field includes more recent publications. Instead, we set a link threshold, that is, we only include publications that have at least two links to other articles. This could be a document that cites one other and is cited by one, or a document that is cited by two other documents in our sample.

Figure 3 below shows how the graph in the main text looks without this 2-link criterion, i.e., if all documents that have at least one link to another document in our sample are included. The separation of the two fields is still very clear, and the conclusions we draw

from Figure 2 in the text are equally valid. What is different are the little "tails" that now spread in different directions. This is exactly what we would expect from a graph that has only a 1-link criterion: One article cites another that in turn also cites only one article, leading to a citation chain rather than a citation cluster.

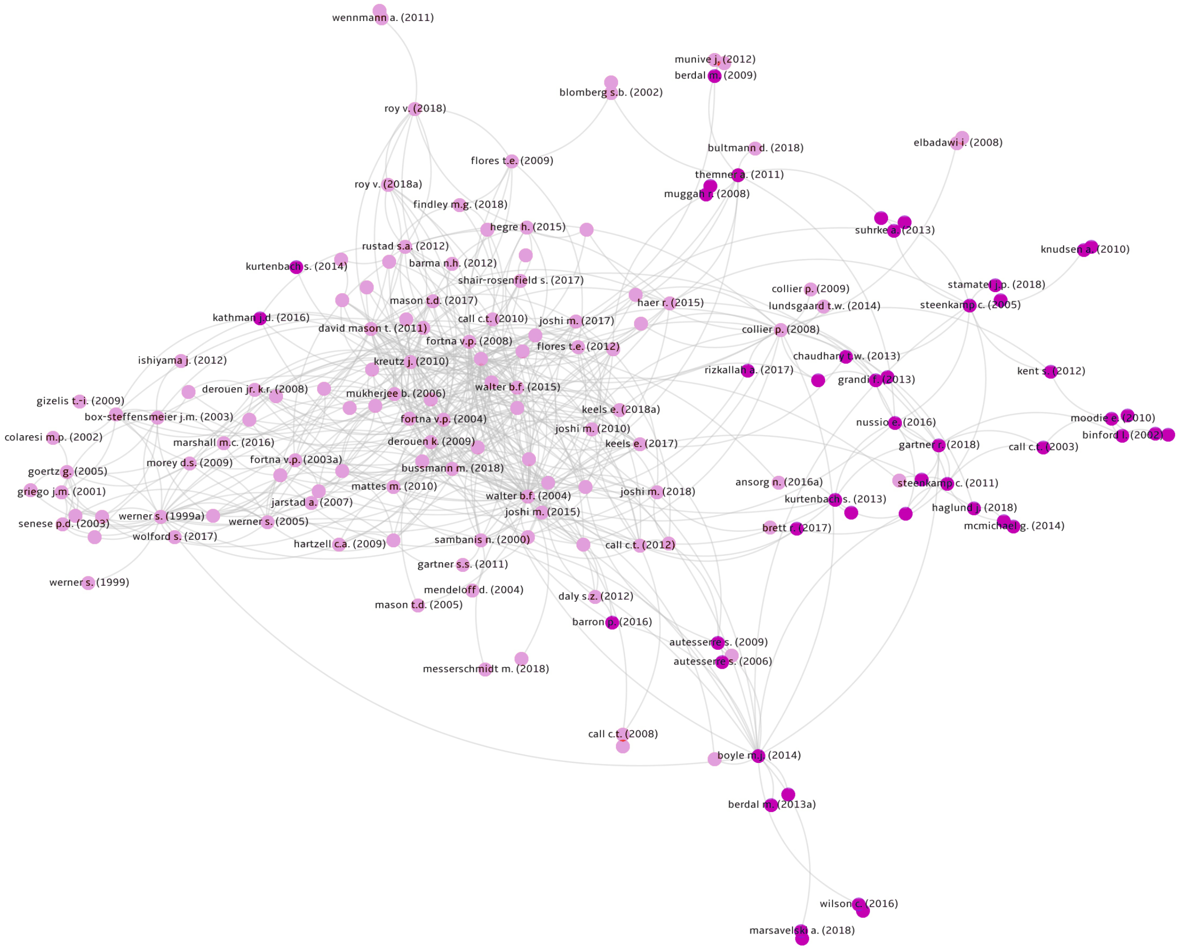

Figure 3: Citation network analysis of research on war recurrence and postwar violence. Included are all publications with one link. Publications that belong to the civil war recurrence literature are coloured in light pink, while publications that belong to the postwar violence literature are coloured in dark pink. Larger circles indicate more links to other documents in the sample.

Full list of references

In graph	Authors	Year	Title	Journal / Book Title	Volume
x	Ansorg N., Haass F., Strasheim J.	2016	Police reforms in peace agreements, 1975–2011: Introducing the PRPA dataset	Journal of Peace Research	53
x	Ansorg N., Kurtenbach S.	2016	Institutional reforms and peacebuilding: Change, path-dependency and societal divisions in pc	Institutional Reforms and Peacebuilding: Change, Path-Depende	N.A.
x	Autesserre S.	2009	Hobbes and the Congo: Frames, local violence, and international intervention	International Organization	63
x	Barron P., Jaffrey S., Varshney A.	2016	When large conflicts subside: The ebbs and flows of violence in post-suharto Indonesia	Journal of East Asian Studies	16
x	Berdal M., Ucko D.H.	2009	Reintegrating armed groups after conflict: Politics, violence and transition	Reintegrating Armed Groups After Conflict: Politics, Violence anc	N.A.
x	Binford L.	2002	Violence in El Salvador: A Rejoinder to Philippe Bourgois's 'The Power of Violence in War and I	Ethnography	3
x	Blomberg S.B., Hess G.D.	2002	The temporal links between conflict and economic activity	Journal of Conflict Resolution	46
x	Box-Steffensmeier J.M., Reiter D., Zorn C.	2003	Nonproportional hazards and event history analysis in international relations	Journal of Conflict Resolution	47
x	Boyle M.J.	2014	Violence after war: Explaining instability in post-conflict states	Violence After War: Explaining Instability in Post-Conflict States	N.A.
x	Braithwaite J.M., Sudduth J.K.	2016	Military purges and the recurrence of civil conflict	Research and Politics	3
x	Brett R.	2017	The Role of Civil Society Actors in Peacemaking: The Case of Guatemala	Journal of Peacebuilding and Development	12
x	Burrell J.L., Moodie E.	2015	The Post-Cold War Anthropology of Central America	Annual Review of Anthropology	44
x	Bussmann M.	2018	Military Integration, Demobilization, and the Recurrence of Civil War	Journal of Intervention and Statebuilding	
x	Call C.T.	2003	Democratisation, war and state-building: Constructing the rule of law in El Salvador	Journal of Latin American Studies	35
x	Call C.T.	2010	Liberia's War Recurrence: Grievance over greed	Civil Wars	12
x	Call C.T.	2012	Why peace fails: The causes and prevention of civil war recurrence	Why Peace Fails: The Causes and Prevention of Civil War Recurre	N.A.
x	Call C.T., Cousens E.M.	2008	Ending wars and building peace: International responses to war-torn societies	International Studies Perspectives	9
x	Cammett M., Malesky E.	2012	Power Sharing in Postconflict Societies: Implications for Peace and Governance	Journal of Conflict Resolution	56
x	Chaudhary T.W.	2013	The political economies of violence in post-war Liberia	The Peace in Between: Post-War Violence and Peacebuilding	N.A.
x	Colaresi M.P., Thompson W.R.	2002	Hot spots or hot hands? Serial crisis behavior, escalating risks, and rivalry	Journal of Politics	64
x	Collier P., Hoeffler A., Söderbom M.	2008	Post-conflict risks	Journal of Peace Research	45
x	Daly S.Z.	2012	Organizational legacies of violence: Conditions favoring insurgency onset in Colombia, 1964-19	Journal of Peace Research	49
x	Daly S.Z.	2014	The dark side of power-sharing: Middle managers and civil war recurrence	Comparative Politics	46
x	Daly S.Z.	2016	Organized violence after civil war: The geography of recruitment in Latin America	Organized Violence after Civil War: The Geography of Recruitme	N.A.
x	David Mason T., Gurses M., Brandt P.T., Michael Quinn J.	2011	When Civil Wars Recur: Conditions for Durable Peace after Civil Wars	International Studies Perspectives	12
x	Deglow A.	2016	Localized legacies of civil war: Postwar violent crime in Northern Ireland	Journal of Peace Research	53
x	DeRouen Jr. K.R., Bercovitch J.	2008	Enduring internal rivalries: A new framework for the study of civil war	Journal of Peace Research	45
x	DeRouen K., Jr., Chowdhury I.	2018	Mediation, Peacekeeping And Civil War Peace Agreements	Defence and Peace Economics	29
x	Derouen K., JR., Lea J., Wallensteen P.	2009	The Duration of Civil War Peace Agreements*	Conflict Management and Peace Science	26
x	Diehl P.F., Reifschneider J., Hensel P.R.	1996	United Nations intervention and recurring conflict	International Organization	50
x	Elbadawi I., Hegre H., Milante G.J.	2008	The aftermath of civil war	Journal of Peace Research	45
x	Faulkner C.M.	2017	Buying Peace? Civil War Peace Duration and Private Military & Security Companies	Civil Wars	
x	Findley M.G.	2018	Does Foreign Aid Build Peace?	Annual Review of Political Science	21
x	Flores T.E., Nooruddin I.	2009	Financing the peace: Evaluating World Bank post-conflict assistance programs	Review of International Organizations	4
x	Flores T.E., Nooruddin I.	2009	Democracy under the gun understanding postconflict economic recovery	Journal of Conflict Resolution	53
x	Flores T.E., Nooruddin I.	2012	The effect of elections on postconflict peace and reconstruction	Journal of Politics	74
x	Fortna V.P.	2003	Inside and out: Peacekeeping and the duration of peace after civil and interstate wars	International Studies Review	5
x	Fortna V.P.	2003	Scraps of paper? Agreements and the durability of peace	International Organization	57
x	Fortna V.P.	2004	Does peacekeeping keep peace? International intervention and the duration of peace after civ	International Studies Quarterly	48
x	Fortna V.P.	2008	Does peacekeeping work?: Shaping belligerents' choices after civil war	Does Peacekeeping Work?: Shaping Belligerents' Choices after C	N.A.
x	Gartner R., Kennedy L.	2018	War and postwar violence	Crime and Justice	47
x	Gartner S.S.	2011	Signs of trouble: Regional organization mediation and civil war agreement durability	Journal of Politics	73
x	Gates S., Graham B.A.T., Lupu Y., Strand H., Strøm K.W.	2016	Power sharing, protection, and peace	Journal of Politics	78
x	Goertz G., Jones B., Diehl P.F.	2005	Maintenance processes in international rivalries	Journal of Conflict Resolution	49
x	Grandi F.	2013	Why do the victors kill the vanquished? Explaining political violence in post-World War II Italy	Journal of Peace Research	50
x	Griego J.M.	2001	Repetitive military challenges and recurrent international conflicts, 1918-1994	International Studies Quarterly	45
x	Gurses M., Rost N.	2013	Sustaining the peace after ethnic civil wars	Conflict Management and Peace Science	30
x	Gurses M., Rost N.	2017	Religion as a Peacemaker? Peace Duration after Ethnic Civil Wars	Politics and Religion	10
x	Gurses M., Rost N., McLeod P.	2008	Mediating civil war settlements and the duration of peace	International Interactions	34
x	Haer R., Böhmelt T.	2015	Child soldiers as time bombs? Adolescents' participation in rebel groups and the recurrence of	European Journal of International Relations	22
x	Haglund J., Richards D.L.	2018	Enforcement of sexual violence law in post-civil conflict societies	Conflict Management and Peace Science	35
x	Hartzell C.A.	2009	Settling Civil Wars: Armed Opponents' Fates and the Duration of the Peace	Conflict Management and Peace Science	26
x	Hegre H., Hultman L., Nygård H.M.	2018	Evaluating the conflict-reducing effect of UN peacekeeping operations	Journal of Politics	

In graph	Authors	Year	Title	Journal / Book Title	Volume
x	Hegre H., Nygård H.M.	2015	Governance and Conflict Relapse	Journal of Conflict Resolution	59
x	Hegre H., Nygård H.M., Ræder R.F.	2017	Evaluating the scope and intensity of the conflict trap: A dynamic simulation approach	Journal of Peace Research	54
x	Herreros F.	2011	Peace of cemeteries: Civil war dynamics in postwar states' repression	Politics and Society	39
x	Howarth K.	2014	Connecting the dots: Liberal peace and post-conflict violence and crime	Progress in Development Studies	14
x	Hultman L., Kathman J.D., Shannon M.	2016	United Nations peacekeeping dynamics and the duration of post-civil conflict peace	Conflict Management and Peace Science	33
x	Ishiyama J., Batta A.	2011	Rebel organizations and conflict management in post-conflict societies 1990-2009	Civil Wars	13
x	Ishiyama J., Breuning M.	2012	Educational Access and Peace Duration in Post-Conflict Countries	International Interactions	38
x	Jarstad A., Sundberg R.	2007	Peace by pact: Data on the implementation of peace agreements	Globalization and Challenges to Building Peace	N.A.
x	Johnson C.	2008	Partitioning to peace: Sovereignty, demography, and ethnic civil wars	International Security	32
x	Joshi M.	2010	Post-civil war democratization: Promotion of democracy in post-civil war states, 1946-2005	Democratization	17
x	Joshi M., Quinn J.M.	2017	Implementing the Peace: The Aggregate Implementation of Comprehensive Peace Agreement	British Journal of Political Science	47
x	Joshi M., Quinn J.M., Regan P.M.	2015	Annualized implementation data on comprehensive intrastate peace accords, 1989–2012	Journal of Peace Research	52
x	Joshi M., Wallensteen P.	2018	Understanding quality peace: Introducing the five dimensions	Understanding Quality Peace: Peacebuilding after Civil War	N.A.
x	Karlen N.	2017	The legacy of foreign patrons: External state support and conflict recurrence	Journal of Peace Research	54
x	Kathman J.D., Wood R.M.	2016	Stopping the Killing During the "Peace": Peacekeeping and the Severity of Postconflict Civilian	Foreign Policy Analysis	12
x	Keels E.	2017	Oil Wealth, Post-conflict Elections, and Postwar Peace Failure	Journal of Conflict Resolution	61
x	Keels E.	2018	Electoral Reforms and Peace Duration Following Negotiated Settlements	International Interactions	44
x	Keels E., Nichols A.D.	2018	State repression and post-conflict peace failure	Conflict, Security and Development	18
x	Kent S., Barnett J.	2012	Localising peace: The young men of Bougainville's 'Crisis generation'	Political Geography	31
x	Knudsen A.	2010	Acquiescence to assassinations in post-civil war Lebanon?	Mediterranean Politics	15
x	Krebs R.R., Licklider R.	2016	United they fall: Why the international community should not promote military integration af	International Security	40
x	Kreutz J.	2010	How and when armed conflicts end: Introducing the UCDP conflict termination dataset	Journal of Peace Research	47
x	Kurtenbach S.	2013	The 'Happy Outcomes' May Not Come at All - Postwar Violence in Central America	Civil Wars	15
x	Letsa N.W.	2017	Voting for peace, mobilizing for war: post-conflict voter turnout and civil war recurrence	Democratization	24
x	Licklider R.	2014	New armies from old: Merging competing militaries after civil wars	New Armies from Old: Merging Competing Militaries after Civil \	N.A.
x	Lo N., Hashimoto B., Reiter D.	2008	Ensuring peace: Foreign-imposed regime change and postwar peace duration, 19142001	International Organization	62
x	Lyons T.	2004	Post-conflict elections and the process of demilitarizing politics: The role of electoral administı	Democratization	11
x	Marsavelski A., Sheremeti F., Braithwaite J.	2018	Did Nonviolent Resistance Fail in Kosovo?	British Journal of Criminology	58
x	Marshall M.C., Ishiyama J.	2016	Does political inclusion of rebel parties promote peace after civil conflict?	Democratization	23
x	Mason T.D., Greig J.M.	2017	State Capacity, Regime Type, and Sustaining the Peace after Civil War	International Interactions	43
x	Mason T.D., Quinn J.	2005	Sustaining the peace: Stopping the recurrence of civil wars	Conflict Prevention and Peace-building in Post-War Societies: Su	N.A.
x	Mattes M., Savun B.	2009	Fostering peace after civil war: Commitment problems and agreement design	International Studies Quarterly	53
x	Mattes M., Savun B.	2010	Information, agreement design, and the durability of civil war settlements	American Journal of Political Science	54
x	Moodie E.	2010	El Salvador in the Aftermath of Peace: Crime, uncertainty, and the transition to democracy	El Salvador in the Aftermath of Peace: Crime, uncertainty, and tł	N.A.
x	Morey D.S.	2009	Conflict and the Duration of Peace in Enduring Internal Rivalries	Conflict Management and Peace Science	26
x	Muggah R.	2008	Security and post-conflict reconstruction: Dealing with fighters in the aftermath of war	Security and Post-Conflict Reconstruction: Dealing with Fighters	N.A.
x	Mukherjee B.	2006	Why political power-sharing agreements lead to enduring peaceful resolution of some civil wa	International Studies Quarterly	50
x	Narang N.	2014	Humanitarian assistance and the duration of peace after civil war	Journal of Politics	76
x	Newman E.	2011	A human security peace-building agenda	Third World Quarterly	32
x	Nussio E., Howe K.	2016	When protection collapses: Post-demobilization trajectories of violence	Terrorism and Political Violence	28
x	Obayashi K.	2018	Distributional effects of political power-sharing arrangements and their negative consequence	Asian Journal of Comparative Politics	3
x	Ohmura H.	2011	Termination and recurrence of civil war: Which outcomes lead to durable peace after civil war	Japanese Journal of Political Science	12
x	Ottmann M., Vüllers J.	2015	The Power-Sharing Event Dataset (PSED): A new dataset on the promises and practices of pow	Conflict Management and Peace Science	32
x	Peterson T.M., Quackenbush S.L.	2010	Not all peace years are created equal: Trade, imposed settlements, and recurrent conflict	International Interactions	36
x	Pospieszna P., Schneider G.	2013	The Illusion of 'Peace Through Power-Sharing': Constitutional Choice in the Shadow of Civil Wa	Civil Wars	15
x	Quackenbush S.L.	2010	Territorial issues and recurrent conflict	Conflict Management and Peace Science	27
x	Quackenbush S.L., Venteicher J.F.	2008	Settlements, outcomes, and the recurrence of conflict	Journal of Peace Research	45
x	Quinn J.M., Mason T.D., Gurses M.	2007	Sustaining the peace: Determinants of civil war recurrence	International Interactions	33
x	Rizkallah A.	2017	The paradox of power-sharing: stability and fragility in postwar Lebanon	Ethnic and Racial Studies	40
x	Rohner D., Thoenig M., Zilibotti F.	2013	War signals: A theory of trade, trust, and conflict	Review of Economic Studies	80
x	Roy V.	2017	Stabilize, rebuild, prevent?: An overview of post-conflict resource management tools	Extractive Industries and Society	4
x	Roy V.	2018	Natural resource production and the risk of conflict recurrence	Studies in Conflict and Terrorism	41
x	Roy V.	2018	Managing Resource-related Conflict: A Framework of Lootable Resource Management and Po:	Journal of Conflict Resolution	62

In graph	Authors	Year	Title	Journal / Book Title	Volume
x	Rudloff P., Findley M.G.	2016	The downstream effects of combatant fragmentation on civil war recurrence	Journal of Peace Research	53
x	Rustad S.A., Binningsbø H.M.	2012	A price worth fighting for? Natural resources and conflict recurrence	Journal of Peace Research	49
x	Sambanis N.	2000	Partition as a solution to ethnic war: An empirical critique of the theoretical literature	World Politics	52
x	Schoon E.W.	2018	Why does armed conflict begin again? A new analytic approach	International Journal of Comparative Sociology	59
x	Schuld M.	2013	The prevalence of violence in post-conflict societies: A case study of KwaZulu-Natal, South Afri	Journal of Peacebuilding and Development	8
x	Senese P.D., Quackenbush S.L.	2003	Sowing the seeds of conflict: The effect of dispute settlements on durations of peace	Journal of Politics	65
x	Shair-Rosenfield S., Wood R.M.	2017	Governing well after war: How improving female representation prolongs post-conflict peace	Journal of Politics	79
x	Steenkamp C.	2005	The legacy of war: Conceptualizing a 'culture of violence' to explain violence after peace accor	Round Table	94
x	Steenkamp C.	2011	In the shadows of war and peace: Making sense of violence after peace accords	Conflict, Security and Development	11
x	Subedi D.B.	2014	Ex-combatants, security and post-conflict violence: Unpacking the experience from Nepal	Millennial Asia	5
x	Suhrke A., Berdal M.	2013	The peace in between: Post-war violence and peacebuilding	The Peace in Between: Post-War Violence and Peacebuilding	N.A.
x	Themner A.	2011	Violence in post-conflict societies: Remarginalization, remobilizers and relationships	Violence in Post-Conflict Societies: Remarginalization, Remobiliz	N.A.
x	Tir J., Diehl P.F.	2002	Geographic dimensions of enduring rivalries	Political Geography	21
x	Toft M.D.	2009	Securing the peace: The durable settlement of civil wars	Securing the Peace: The Durable Settlement of Civil Wars	N.A.
x	Uzonyi G., Hanania R.	2017	Government-sponsored mass killing and civil war reoccurrence	International Studies Quarterly	61
x	Walter B.F.	2004	Does conflict beget conflict? Explaining recurring civil war	Journal of Peace Research	41
x	Walter B.F.	2015	Why Bad Governance Leads to Repeat Civil War	Journal of Conflict Resolution	59
x	Wanis-St. John A., Kew D.	2008	Civil society and peace negotiations: Confronting exclusion	International Negotiation	13
x	Wennmann A.	2011	Breaking the conflict trap? addressing the resource curse in peace processes	Global Governance	17
x	Werner S.	1999	The precarious nature of peace: Resolving the issues, enforcing the settlement, and renegotiat	American Journal of Political Science	43
x	Werner S., Yuen A.	2005	Making and keeping peace	International Organization	59
x	Wilson C.	2016	Ongoing rebel violence in autonomous regions: Assam, Northeast India	Pacific Affairs	89
x	Wolford S.	2017	The problem of shared victory: War-winning coalitions and postwar peace	Journal of Politics	79
x	Zeigler S.M.	2016	Competitive alliances and civil war recurrence	International Studies Quarterly	60
	Amneus D.	2011	Insufficient legal protection and access to justice for post-conflict sexual violence	Development Dialogue	
	Autesserre S.	2006	Local Violence, National Peace? Postwar "Settlement" in the Eastern D.R. Congo (2003–2006)	African Studies Review	49
	Autesserre S.	2016	The responsibility to protect in Congo: The failure of grassroots prevention	International Peacekeeping	23
	Bakke K.M., Linke A.M., O'Loughlin J., Toal G.	2018	Dynamics of state-building after war: External-internal relations in Eurasian de facto states	Political Geography	63
	Barma N.H.	2012	Peace-building and the predatory political economy of insecurity: Evidence from Cambodia, Ea	Conflict, Security and Development	12
	Baytiyeh H.	2018	Education to Reduce Recurring Conflicts	Peace Review	30
	Bedoya J.J.	2017	Coercive social extortion and the miracle of Medellin: The counter face of a model [La coerciór	Canadian Journal of Latin American and Caribbean Studies	42
	Ben D.M., Maoz Z.	1999	Learning and the evolution of enduring international rivalries: A strategic approach	Conflict Management and Peace Science	17
	Benedek W.	2010	The human security approach to terrorism and organized crime in post-conflict situations	Transnational Terrorism, Organized Crime and Peace-Building: H	N.A.
	Benson P., Fischer E.F., Thomas K.	2008	Resocializing suffering: Neoliberalism, accusation, and the sociopolitical context of guatemala'	Latin American Perspectives	35
	Berdal M.	2013	Reflections on post-war violence and peacebuilding	The Peace in Between: Post-War Violence and Peacebuilding	N.A.
	Berdal M., Collantes-Celador G., Buzadzic M.Z.	2013	Post-war violence in Bosnia and herzegovina	The Peace in Between: Post-War Violence and Peacebuilding	N.A.
	Berend I.T.	2006	Editorial: The Kosovo trap	European Review	14
	Beyerle S.	2011	Civil resistance and the corruption-violence nexus	Journal of Sociology and Social Welfare	38
	Bilukha O.O., Becknell K., Laurenge H., Danee L., Subedi K.P.	2013	Fatal and non-fatal injuries due to intentional explosions in Nepal, 2008-2011: Analysis of surv	Conflict and Health	7
	Borer T.A.	2009	Gendered war and gendered peace: Truth Commissions and postconflict gender violence: Less	Violence Against Women	15
	Bowsher G., Bogue P., Patel P., Boyle P., Sullivan R.	2018	Small and light arms violence reduction as a public health measure: The case of Libya	Conflict and Health	12
	Bradley S.	2018	Domestic and Family Violence in Post-Conflict Communities: International Human Rights Law a	Health and human rights	20
	Braithwaite A., Dasandi N., Hudson D.	2016	Does poverty cause conflict? Isolating the causal origins of the conflict trap	Conflict Management and Peace Science	33
	Broadhurst R.	2002	Lethal violence, crime and state formation in Cambodia	Australian and New Zealand Journal of Criminology	35
	Bultmann D.	2018	Insurgent Groups During Post-Conflict Transformation: The Case of Military Strongmen in Cam	Civil Wars	20
	Buzard K., Graham B.A.T., Horne B.	2017	Unrecognized states: A theory of self-determination and foreign influence	Journal of Law, Economics, and Organization	33
	Cardenas E., Gleditsch K.S., Guevara L.C.	2018	Network structure of insurgent groups and the success of DDR processes in Colombia	Small Wars and Insurgencies	29
	Cardoso L.F., Gupta J., Shuman S., Cole H., Kpebo D., Falb K.L.	2016	What Factors Contribute to Intimate Partner Violence Against Women in Urban, Conflict-Affec	Journal of Urban Health	93
	Carey Jr. D., Torres M.G.	2010	Precursors to femicide: Guatemalan women in a vortex of violence	Latin American Research Review	45
	Cevik S., Rahmati M.	2015	Breaking the Curse of Sisyphus: An Empirical Analysis of Post-Conflict Economic Transitions	Comparative Economic Studies	57
	Childers J.	2014	Amnesty, revenge, and the threat of conflict relapse	International Criminal Law Review	14
	Collier P., Chauvet L., Hegre H.	2009	The security challenge in conflict-prone countries	Global Crises, Global Solutions	N.A.
	Cornell S., Jonsson M.	2014	Conflict, crime, and the state in postcommunist Eurasia	Conflict, Crime, and the State in Postcommunist Eurasia	N.A.

In graph	Authors	Year	Title	Journal / Book Title	Volume
	Cusato E.	2017	Back to the Future? Confronting the Role(s) of Natural Resources in Armed Conflict Through th	International Community Law Review	19
	Darch C.	2016	Separatist tensions and violence in the ‘model post-conflict state’: Mozambique since the 199	Review of African Political Economy	43
	Das G.	2012	Security and Development in India's Northeast	Security and Development in India's Northeast	N.A.
	De La Calle L.	2015	Nationalist violence in postwar Europe	Nationalist Violence in Postwar Europe	N.A.
	del Castillo G., de Soto Á.	2017	Obstacles to peacebuilding	Obstacles to Peacebuilding	N.A.
	Demeritt J.H.R., Nichols A.D., Kelly E.G.	2014	Female Participation and Civil War Relapse	Civil Wars	16
	Diepart J.-C., Dupuis D.	2014	The peasants in turmoil: Khmer Rouge, state formation and the control of land in northwest C	Journal of Peasant Studies	41
	Du Toit L.	2014	Shifting meanings of postconflict sexual violence in South Africa	Signs	40
	El-Bushra J.	2017	How should we explain the recurrence of violent conflict, and what might gender have to do w	The Oxford Handbook of Gender and Conflict	N.A.
	Elliott C.V., Elliott V.L.	2005	The economics of sustaining the peace: Breaking the Conflict Trap - civil war and development	Conflict Prevention and Peace-building in Post-War Societies: Su	N.A.
	Enria L.	2015	Love and Betrayal: The Political Economy of Youth Violence in Post-War Sierra Leone	Journal of Modern African Studies	53
	Farrall J.	2013	Recurring dilemmas in a recurring conflict: Evaluating the un mission in Liberia (2003-2006)	Peace Operation Success: A Comparative Analysis	N.A.
	Gizelis T.-I.	2009	Gender empowerment and United Nations peacebuilding	Journal of Peace Research	46
	Gobodo-Madikizela P., Fish J., Shefer T.	2014	Gendered violence: Continuities and transformation in the aftermath of conflict in Africa	Signs	40
	Godec S.T.	2010	Between rhetoric and reality: Exploring the impact of military humanitarian intervention upon	International Review of the Red Cross	92
	Guneylioglu M.	2017	War, Status Quo, and Peace in the South Caucasus: A Power Transition Perspective	Public Integrity	19
	Hensel P.R.	1996	Charting a course to conflict: Territorial issues and interstate conflict, 1816-1992	Conflict Management and Peace Science	15
	Hoglund K., Orjuela C.	2011	Winning the peace: Conflict prevention after a victor’s peace in sri lanka	Contemporary Social Science	6
	Holland C., Rabrenovic G.	2017	Social Immobility, Ethno-politics, and Sectarian Violence: Obstacles to Post-conflict Reconstruc	International Journal of Politics, Culture and Society	30
	Ishiyama J.	2016	Introduction to the special issue “From bullets to ballots: the transformation of rebel groups in	Democratization	23
	Jesperson S.	2016	Rethinking the security-development nexus: Organised crime in post-conflict states	Rethinking the Security-Development Nexus: Organised Crime in	N.A.
	Joshi M.	2014	Post-Accord Political Violence, Elections, and Peace Processes: Evidence from Nepal	Civil Wars	16
	Khan A.	2009	Renewed ethnonationalist insurgency in balochistan, pakistan: The militarized state and contin	Asian Survey	49
	Kibris A.	2015	The Conflict Trap Revisited: Civil Conflict and Educational Achievement	Journal of Conflict Resolution	59
	Knudsen A., Yassin N.	2013	Political violence in post-civil war Lebanon	The Peace in Between: Post-War Violence and Peacebuilding	N.A.
	Kreutz J., Marsh N., Torre M.	2013	Regaining state control: Arms and violence in post-conflict countries	Small Arms, Crime and Conflict: Global Governance and the Thre	N.A.
	Kurtenbach S.	2014	Postwar violence in Guatemala: A mirror of the relationship between youth and adult society	International Journal of Conflict and Violence	8
	Lalic V., Duric S.	2018	Policing hate crimes in Bosnia and Herzegovina	Policing and Society	28
	Le Billon P., Savage E.	2016	Binding pipelines? Oil, armed conflicts, and economic rationales for peace in the two Sudans	African Geographical Review	35
	Le Huerou A.	2014	Between war experience and ordinary police rationales: State violence against civilians in post	Chechnya at War and Beyond	N.A.
	Little W.E.	2009	Living and selling in the "new violence" of guatemala	Mayas in Postwar Guatemala: Harvest of Violence Revisited	N.A.
	Llorente M.V.	2015	From War to peace: Security and the stabilization of Colombia	Stability	4
	Long W.J., Brecke P.	2003	The emotive causes of recurrent international conflicts	Politics and the Life Sciences	22
	Lounsbery M.O., Cook A.H.	2011	Rebellion, mediation, and group change: An empirical investigation of competing hypotheses	Journal of Peace Research	48
	Lovell W.G.	2010	A beauty that hurts: Life and death in Guatemela, Second revised edition	A Beauty That Hurts: Life and Death in Guatemela, Second Revis	N.A.
	Lundsgaard T.W.	2014	Peace for sale: What is the role of human rights-based CSR in the extractive industry in post-co	Business, Peace and Sustainable Development	2014
	Manz B.	2008	The continuum of violence in post-war Guatemala	Social Analysis	52
	McMichael G.	2014	Rethinking access to land and violence in post-war cities: Reflections from Juba, Southern Sud	Environment and Urbanization	26
	McNeish J.-A., Rivera O.L.	2009	The ugly poetics of violence in post-accord Guatemala	Forum for Development Studies	36
	McNeish J.-A., Rivera O.L.	2013	The multiple forms of violence in post-war Guatemala	The Peace in Between: Post-War Violence and Peacebuilding	N.A.
	Medie P.A.	2018	The police: Laws, prosecutions and women’s rights in Liberia	Institutions and Democracy in Africa: How the Rules of the Game	N.A.
	Mendeloff D.	2004	Truth-seeking, truth-telling, and postconflict peacebuilding: Curb the enthusiasm?	International Studies Review	6
	Messerschmidt M.	2018	Ingrained practices: Sexual violence, hypermasculinity, and re-mobilisation for violent conflict	Global Society	32
	Moncrief S.	2017	Military socialization, disciplinary culture, and sexual violence in UN peacekeeping operations	Journal of Peace Research	54
	Muggah R., Krause K.	2011	Closing the gap between peace operations and post-conflict insecurity: Towards a violence-red	Peace Operations and Organized Crime: Enemies or Allies?	N.A.
	Munive J., Jakobsen S.F.	2012	Revisiting DDR in Liberia: Exploring the power, agency and interests of local and international	Conflict, Security and Development	12
	Munive J., Stepputat F.	2015	Rethinking disarmament, demobilization and reintegration programs	Stability	4
	Nakaya S.	2009	Aid and transition from a war economy to an oligarchy in post-war Tajikistan	Central Asian Survey	28
	Novelli M., Higgins S.	2017	The violence of peace and the role of education: insights from Sierra Leone	Compare	47
	O'Dowd L., McKnight M.	2013	Urban Intersections: Religion and Violence in Belfast	Space and Polity	17
	Pankhurst D.	2012	Post-war backlash violence against women: What can “masculinity” explain?	Gendered Peace: Women's Struggles for Post-War Justice and Re	N.A.
	Peou S.	2013	Violence in post-war Cambodia	The Peace in Between: Post-War Violence and Peacebuilding	N.A.
	Piccolino G.	2016	Conference report: The legacy of armed conflicts: Southern African and comparative perspecti	Africa Spectrum	51

In graph	Authors	Year	Title	Journal / Book Title	Volume
	Proksik J.J.	2013	Organized Crime and the Dilemmas of Democratic Peace-Building in Kosovo	International Peacekeeping	20
	Ramisetty A., Muriu M.	2013	'When does the end begin?' Addressing gender-based violence in post-conflict societies: Case	Gender and Development	21
	Rampton D.	2011	'Deeper hegemony: The politics of Sinhala nationalist authenticity and the failures of power-sh	Commonwealth and Comparative Politics	49
	Reuveny R.	2000	Resource scarcity and conflict in developing countries	Journal of Peace Research	37
	Reynolds J.F.	2013	Refracting articulations of citizenship, delicuencia and vigilantism in boys' sociodramatic play i	Language and Communication	33
	Richani N.	2010	State Capacity in Postconflict Settings: Explaining criminal violence in El Salvador and Guatema	Civil Wars	12
	Richmond O.P.	2012	A Pedagogy of Peacebuilding: Infrapolitics, Resistance, and Liberation	International Political Sociology	6
	Saikia A.	2012	The historical geography of the Assam violence	Economic and Political Weekly	47
	Saile R., Neuner F., Ertl V., Catani C.	2013	Prevalence and predictors of partner violence against women in the aftermath of war: A surve	Social Science and Medicine	86
	Sandole D.J.D., Staroste I.	2015	Making the Case for Systematic, Gender-Based Analysis in Sustainable Peace Building	Conflict Resolution Quarterly	33
	Sandoval Giron A.B.	2007	Taking matters into one's hands: Lynching and violence in post-civil war Guatemala	Urban Anthropology	36
	Santiso C.	2002	Promoting democractic governance and preventing the recurrence of conflict: The role of the	Journal of Latin American Studies	34
	Schroeder U.C., Friesendorf C.	2009	State-building and organized crime: Implementing the international law enforcement agenda	Journal of International Relations and Development	12
	Scully P.	2014	Development and its discontents: Ending violence against women in post-conflict Liberia	Sexual Violence in Conflict and Post-Conflict Societies: Internatio	N.A.
	Sengupta A., Calo M.	2016	Shifting gender roles: an analysis of violence against women in post-conflict Uganda	Development in Practice	26
	Sharp E.	2012	Between justice and vigilantism: Organizing to confront crime in post-war guatemala	Grassroots Development	33
	Silber I.C.	2004	Mothers/fighters/citizens: Violence and disillusionment in post-war El Salvador	Gender and History	16
	Sisk T.D.	2008	Peacebuilding as democratization: Findings and recommendations	From War to Democracy: Dilemmas of Peacebuilding	N.A.
	Smith T.J., Offit T.A.	2010	Confronting violence in postwar guatemala: An introduction	Journal of Latin American and Caribbean Anthropology	15
	Springer S.	2010	Cambodia's neoliberal order: Violence, authoritarianism, and the contestation of public space	Cambodia's Neoliberal Order: Violence, Authoritarianism, and th	N.A.
	Stamatel J.P., Romans S.H.	2018	The Effects of Wars on Postwar Homicide Rates: A Replication and Extension of Archer and Ga	Journal of Contemporary Criminal Justice	34
	Steenkamp C.	2017	The crime-conflict nexus and the civil war in Syria	Stability	6
	Suhardiman D., Rutherford J., Bright S.J.	2017	Putting violent armed conflict in the center of the Salween hydropower debates	Critical Asian Studies	49
	Thornhill K.	2012	"You must sit on the old mat to ply the new one": Rethinking threatened masculinities and po	Engaging Men in the Fight against Gender Violence: Case Studies	N.A.
	Toupin S.	2014	Women peacekeepers and UNPOL officers in the fight against sexual and gender-based violen	Sexual Violence in Conflict and Post-Conflict Societies: Internatio	N.A.
	True J.	2018	The political economy of post-conflict violence against women	Handbook on the International Political Economy of Gender	N.A.
	Turshen M.	2016	Gender and the political economy of conflict in Africa: The persistence of violence	Gender and the Political Economy of Conflict in Africa: The Persis	N.A.
	Tyner J.A., Devadoss C.	2014	Administrative violence, prison geographies and the photographs of Tuol Sleng Security Cente	Area	46
	Tyner J.A., Will R.	2015	Nature and post-conflict violence: water management under the Communist Party of Kampuc	Transactions of the Institute of British Geographers	40
	van Baalen S., Hoglund K.	2017	“So, the Killings Continued”: Wartime Mobilization and Post-War Violence in KwaZulu-Natal, S	Terrorism and Political Violence	
	Wennmann A.	2005	Resourcing the recurrence of intrastate conflict: Parallel economies and their implications for	Security Dialogue	36
	Werner S.	1999	Choosing demands strategically: The distribution of power, the distribution of benefits, and th	Journal of Conflict Resolution	43
	Willcoxon G.F.	2017	Contention, Violence and Stalemate in Post-War Libya	Mediterranean Politics	22
	Williams P.	2011	Criminals, militias, and insurgents: Organized crime in Iraq	Crime and Insurgency in Iraq and Afghanistan	N.A.
	Wilson C.	2017	Ideological Motives in Spoiler Violence: Postconflict Assam, Northeast India	Nationalism and Ethnic Politics	23
	Winton A.	2004	Young people's views on how to tackle gang violence in " post-conflict" Guatemala	Environment and Urbanization	16
